# Cherokee County Medical Society

**Published:** 1888-11

**Authors:** J. H. Evans

**Affiliations:** Acting Secretary


### Society Notes.

### CHEROKEE COUNTY MEDICAL SOCIETY.

Reported for Daniel's Texas Medical Journal by J. H. Evans, M. D., Acting Secretary.

The Cherokee County Medical Society held its third quarterly meeting at Jacksonville, October 2 and 3, 1888, the president, Dr. A. H. McCord, in the chair, and in the absence of the secretary, Dr. Johnson, the assistant secretary, Dr. J. H. Evans, acted. The attendance was larger than usual, and much interest manifested. Several very interesting papers were read, which led to a lively discussion, besides several clinical reports of cases.

The object of this association is the consideration of scientific medicine, and the cultivation of a closer relationship between the medical fraternity. The subjects for next meeting, which will be held at Rusk, Texas, the first Tuesday in January, 1889, are as follows: "Catarrhal Fever," a paper by Dr. J. K. Frazier, of Rusk, Texas; "Croup," a paper by Dr. Johnson, of the same place; "Pneumonia," a paper by Dr. John B. Guinn, of Alto, Texas. The committee of collective investigation will report, besides all are requested to make clinical reports of cases. We anticipate another good meeting in January. Territory limited to this connty, and membership is free to all.

Alto, Texas, Oct. 30, 1888.

### REPORT OF SPECIAL COMMITTEE

On Collective Investigation of Disease, before the Cherokee County Medical Association, sent for publication by vote of the Society, to Daniel's Texas Medical Joournal:

The following is submitted as a committee report on collective investigation of disease:

We having selected as a subject for our data collection the much agitated question and unfortunate offspring of two kingdoms, typho-malarial fever, propounded nine interrogatories, and sent same to the physicians of the county—thirty-three in all. In answer to these questions, twenty replies have been received.

From those answers we glean the following: To question first, "Has typho-malarial fever been very prevalent in your practice during the past twelve months?" ten answer, "not very prevalent," or "less than in former years;" four "prevalent." A noteworthy fact with reference to those four physicians is, they all practice in very malarial districts; one in the swamps of the Angelina river, another towards the slopes of the Neches river, another along one of the largest tributaries of the last named river, while the fourth one received the most of his cases from convict camps.

Second. "Has there been more or less typho-malarial fever than of former years?" Nine answer "less," four "more," two "about the same," three gave no answer. The same four who answered the first question in the affirmative are the four who have had more of said fever to contend with in the past twelve months than in former years.

To question three, "Has the fever been attended with much fatality?" fourteen answer "it has not," one "yes," one lost one in three cases that he had had; three gave no reply to the question.

Fourth. "During what months has the fever prevailed mostly?" While every month in the calendar is drawn upon to pay tribute to this disease, there is almost a unit in including the months of September and October. Fourteen name those two months in their enumerations; others include them in the terms, "during the autumn months," "through the fall months," etc.

Fifth. "As a rule, which symptoms predominate, the typhoid or the malarial?" Twelve answer the "typhoid," five the "malarial," two "symptoms about equal," one not heard from.

Sixth. "What per cent. of cases have the well-marked enteric symptoms of typhoid?" To this question there is a greater diversity of answers than to any other.

There is a wide, a striking discrepency in the figures in answer to this question, the two extremes being o per cent. and

100 per cent. The graduation in the ascent of this pathological chain of morbid entities is, two say "none," one "2 per cent.," two "10 per cent.," one "25 per cent.," one "50 per cent.," one "66⅔ per cent.," two "75 per cent.," one "83⅓ per cent.," one "100 per cent."

Seventh. "Do you consider typho-malarial fever a disease *per se*, or a mixed one?" In answer to this there are some spicy, racy conjectures, some cogent reasoning, some unique thoughts. Eight claim it to be a mixed disease, six, a disease "by itself"—*per se*; one, typhoid; one, a hybrid; four claim there is a factor generated *de novo*, which produces the typhoid *state*, for such they consider it,—a typhoid condition, and not typhoid fever proper. These four contend that there is following the deleterious inroads of the malarial germ—which exists for the first few days of the disease—a new element, or rather a ferment is generated from the *debris* or tissue waste—caused by an overdose (so to speak) of the Palmelic spores. They claim that there is a considerable amount of effete matter, or necrobiotic detritus thrown into the circulation to empoison the living tissues, and by the fermentative decomposition of these retrograde tissue products the functional activity of the organs of secretion and elimination are so vitiated and impaired as to produce the adynamic condition, with all the symtomatic phenomena of the disease under consideration.

In a normal condition of the animal economy the functional activity of the excretory or eliminative organs and those of absorption, assimilation and resorption is only commensurate with the expenditure of vital energy. Then destroy this equilibrium —this organic functional balance between alimentation and effete elimination, and you have moulded the first link of a pathological chain of morbid sequences. Destroy the functional activity of the hepatic cells and you have derangement of the *prima viæ* with a deleterious reflex throughout the whole system. Tip the balance of power in the malpighian corpuscles of the kidneys and uræmia or its opposite, polydipsia, with the onus of the morbid reflex on the nervous system will be the result; and so on. Then may not this adynamic—this typhoid condition of the socalled tvpho-malarial fever be accounted for by a great quantity of nercobiotic detritus being set free in the vital current, thereby destroying the functional balance above mentioned? Hence the low ebb of the vital forces, while the slow and tedious process of elimination is going on; hence the rational drift of the profession in the management of the trouble—as little medicine as possible to meet indications, supporting measures, food easily digested liquid food, that it may be readily assimilated.

Eighth. "Does quinine arrest or even exercise a controlling influence in this fever?" The answers to this are almost a unit in condemning the use of quinine in large doses after the first four or five days—after the malarial element has been destroyed; four "give it as a tonic" throughout the whole course of the disease; one "thinks it is probably indicated but twice during the twenty-four hours," that is "at 2 o'clock at night and just before day," for fear, if given at any other hour, "it might rupture a blood vessel in the brain."

Ninth. "What is your treatment for said fever, including dietetics?" The treatment of eighteen out of twenty is fully justified by their answer to question three. There is seldom seen such unanimity of expression with regard both to the use of medicine and nourishment, as there is in answer to question nine: "Quinine and mercury" for the first four days to "eliminate the malarial element;" then "tonic and supporting measures, with good, rich, generous, easily digested liquid food, and meet indications as they arise." While different physicians used different drugs, yet in the main the physiological action was the same, 2 gr. quinine, creosote, carbolic acid, iodide potash, Fowler's solution arsenic, etc. Four claimed unusually good results from a combination of turpentine emulsion, tinct. digitalis and Fowler's solution arsenic.

Respectfully,

W. G. JAMISON,<br>

J. K. FRAZER, } Committee.<br>

A. H. MCCORD.

